# Study of Electronic Structure, Thermal Conductivity, Elastic and Optical Properties of α, β, γ-Graphyne

**DOI:** 10.3390/ma11020188

**Published:** 2018-01-25

**Authors:** Xun Hou, Zhongjing Xie, Chunmei Li, Guannan Li, Zhiqian Chen

**Affiliations:** Faculty of Materials and Energy, Southwest University, Chongqing 400715, China; hxfighting@email.swu.edu.cn (X.H.); xzjlms13@email.swu.edu.cn (Z.X.); lcm1998@swu.edu.cn (C.L.); liguannan@swu.edu.cn (G.L.)

**Keywords:** graphyne, electronic structures, elasticity, optical properties

## Abstract

In recent years, graphyne was found to be the only 2D carbon material that has both sp and sp^2^ hybridization. It has received significant attention because of its great potential in the field of optoelectronics, which arises due to its small band gap. In this study, the structural stability, electronic structure, elasticity, thermal conductivity and optical properties of α, β, γ-graphynes were investigated using density functional theory (DFT) systematically. γ-graphyne has the largest negative cohesive energy and thus the most stable structure, while the β-graphyne comes 2nd. Both β and γ-graphynes have sp-sp, sp-sp^2^ and sp^2^-sp^2^ hybridization bonds, of which γ-graphyne has shorter bond lengths and thus larger Young’s modulus. Due to the difference in acetylenic bond in the structure cell, the effect of strain on the electronic structure varies between graphynes: α-graphyne has no band gap and is insensitive to strain; β-graphyne’s band gap has a sharp up-turn at 10% strain, while γ-graphyne’s band gap goes up linearly with the strain. All the three graphynes exhibit large free carrier concentration and these free carriers have small effective mass, and both free carrier absorption and intrinsic absorption are found in the light absorption. Based on the effect of strain, optical properties of three structures are also analyzed. It is found that the strain has significant impacts on their optical properties. In summary, band gap, thermal conductivity, elasticity and optical properties of graphyne could all be tailored with adjustment on the amount of acetylenic bonds in the structure cell.

## 1. Introduction

Some 80 years ago, Peierls and Landau, who researched thermodynamic stability in low-dimensional materials, found the displacement of atoms in low dimension materials by the lattice wave during the heat dissipation process, and the resultant lattice distortion and increase in free energy make the existence of 2D material highly unlikely in reality [1,2]. Subsequently, Mermin’s experiment shows a drop in the material’s melting point with a decrease of its thickness, and at several dozens of atom layers, the material is already thermal-dynamically instable [3]. This theory was well accepted for almost a century, until in 2004, Novoselov K.S. and Geim A.K. at the University of Manchester successfully peeled off from a bulk graphite a single atom layer graphene sheet [4,5].

Graphene is an allotrope of carbon in the form of a two-dimensional, atomic-scale, honeycomb lattice. It is the basic structural element of other allotropes including 0D fullerenes, 1D carbon nanotubes as well as 3D bulk materials [6]. The half-metallic graphene with its unique electronic structure and properties has been regarded as a promising new material [7,8,9,10,11]. There are two Dirac cones with opposite directions in the band structure, with the valence band and conduction band coinciding at the Fermi level to form a Dirac point, which means graphene is actually a semiconductor with zero band gap [12,13]. The special properties of graphene that are different from that of bulk materials have aroused interest in the synthesis and exploration of other two-dimensional materials.

In 2010, by cross-coupling reaction on copper surface, Yuliang Li et al. synthesized a new 2D carbon allotrope graphdiyne successfully [14]. Usually, carbon materials’ dimensionality depends on its hybridization [15]. For example, sp^2^ hybridization leads to 2D graphene, while sp and sp^3^ hybridization form 1D and 3D carbon materials, respectively [16,17]. However, graphyne is found to be the only 2D carbon material so far that has both sp and sp^2^ hybridization [18], which gives rise to its versatile and flexible crystal structures. Particularly, an acetylenic bond formed with sp hybridization, with its versatile space configuration, could give rise to many graphyne structures, among which α, β and γ structures are explored the most. The presence of the acetylenic bond brings about peculiar electronic structures. Different from zero-gap graphene, there is a small gap in graphyne’s Brillouin zone [19,20]. Under room temperature, carriers in graphyne exhibit high mobility. Like graphene, graphyne also has Dirac cones with opposite directions in its energy band, but the band gap increases with the number of acetylenic bond in a single cell, which is not the case in graphene. This band gap controllability offers great application potentials [14]. The unique characteristics of graphyne have attracted research attention [13,21,22]. 

Recently, graphynes have been candidates for application as ultraviolet light protectors [23], transistors [24], catalysts [25], energy storage materials [26,27]. In order to shed light on these applications, the electronic structures [28,29,30,31], mechanical properties [27,32,33], optical properties [19,34] and thermal conductivity at room temperature [35] were explored through the first-principles. However, the effects of strain on optical properties and the minimum thermal conductivity at high temperature of three materials are still missing. Little has been reported on their properties systematically. In this paper, based on DFT (the first-principles), calculation is carried out on α, β, γ-graphynes to study their electronic structure, elasticity, the minimum thermal conductivity and optical properties. Considering these previous studies, a full and detailed systematic analysis of the effect of strain on the band structure and optics of graphyne are still missing. Therefore, it is of great practical significance to conduct a comprehensive and systematic study of the photoelectric properties of the system by using accurate calculation methods. This article will provide a full analysis of the effect of strain on the band structures. The calculation methods as well as parameters are introduced in the second part. The third part provides the analysis on electronic structure, stability, elasticity, thermal conductivity and optical properties, while the conclusion is drawn in the fourth part.

## 2. Calculation and Models

The calculations were performed with CASTEP (Cambridge Sequential Total Energy Package) software package in MS (Material Studio) (MS 6.0 version which was developed by Accelrys Company of America was used) based on the first-principles of DFT [36]. The wave function is expanded along the plane wave basic vector with the crystal boundary condition. Generalized gradient approximation (GGA) of the Perdew-Burke-Ernzerhof (PBE) approach was used to describe the exchange correlation functional [37]. The potential of ion core-valence electron interaction was described by Ultrasoft Pseudopotential (USPP) [38], which took 2s^2^2p^2^ as the outer-shell electron configuration. In particular, DFT-D approaches to treat long-range dispersion correction were employed, notably the Grimme [39] correction to PBE. According to the convergence test, the plane wave cut-off energy was 500 eV throughout our calculation. Each calculation was considered to be converged when the total energy changes during the geometry optimization process were smaller than 5 × 10^−6^ eV/atom and the forces per atom were reduced to 0.01 eV/Å. The residual stress of unit cell was below 0.05 GPa, and the maximum displacement between cycles was less than 0.001 Å when the convergence was reached. The vacuum layer thickness was set to 10 Å to eliminate the interaction between layers. The calculations in the first irreducible Brillouin zone were conducted with different k point meshes using the Monkhorst-Pack scheme [40]. For α and γ-graphyne, 11 × 11 × 1 was picked as the high symmetry K point for Brillouin-zone integration, while for β-graphyne model it is 7 × 11 × 1. Self-consistent iteration for structural optimization was carried out [41] before the calculation and analysis of the electronic structure. As the strain tended to occur in the x and y direction to give rise to raised energy level, in elasticity calculation 7 × 11 × 1 was selected as the high symmetry K point for Brillouin-zone integration for α and γ-graphynes, and 6 × 8 × 1 for β-graphyne. 

**Figure 1:**
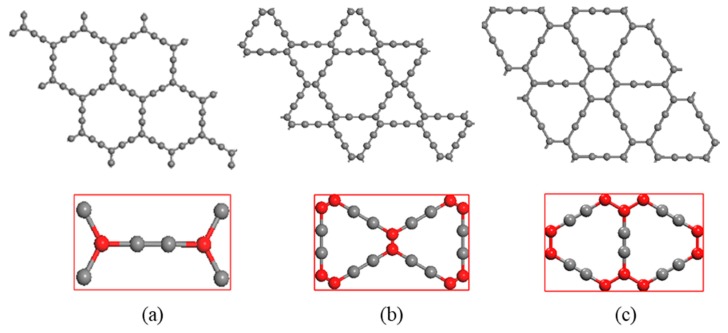
Three crystal structures of graphyne (**a**) α-graphyne (**b**) β-graphyne (**c**) γ-graphyne.

The models for study are α, β and γ-graphyne, whose super cell structures are shown in Figure 1, in which the unit cells are the rectangles marked red. The unit cells of α, β, γ-graphyne consist of 8, 18 and 12 carbon atoms respectively. The red and gray balls represent carbon atoms with sp^2^ and sp hybridization, respectively. Two sp hybridized carbon atoms form an acetylenic bond, and the number and position of acetylenic bonds may vary to give rise of versatile graphyne crystal structures, of which the cavity radius also varies. 

The lattice constants of the optimal unit cell are given in Table 1. These constants are in good agreement with previous works. Both sp and sp^2^ hybridization are found in graphyne, and they form different bonds: sp^2^ for σ and π bonds, and sp for acetylenic bond. The bond particulars (type and length) in α, β, γ-graphynes are also shown in Table 1 (the sp-sp^2^ hybridization bond is missing in α-graphyne).

To evaluate the thermodynamic equilibrium of three structure, cohesive energy per atom ($E_{coh}$) was calculated in this work. Stability of graphyne is gauged with the cohesive energy between its carbon atoms, which is equivalent to the energy required for the separation of the neutral atoms in the ground state at 0 K [42]. Cohesive energy of compounds is the energy released by binding atoms to crystals. A larger negative value indicates that the compound is more stable. Its calculation formula is given as [43]:

(1) $$E_{coh}=\left(E_{total}-n\times E_{atom}\right)/n$$

where *n* is the number of carbon atoms in a unite cell, *E_atom_* is the single atom energy and *E_total_* the total energy of the unit cell.

As listed in Table 1, the calculated cohesive energy of three materials are negative values, indicating that they are all stable at ambient conditions. The γ-graphyne has the largest negative cohesive energy, which means that it is the most stable. The stability, in descending order, of the three graphynes is γ-graphyne, β-graphyne and α-graphyne. 

## 3. Results and Discussion

### 3.1. Electronic Structures

**Figure 2:**
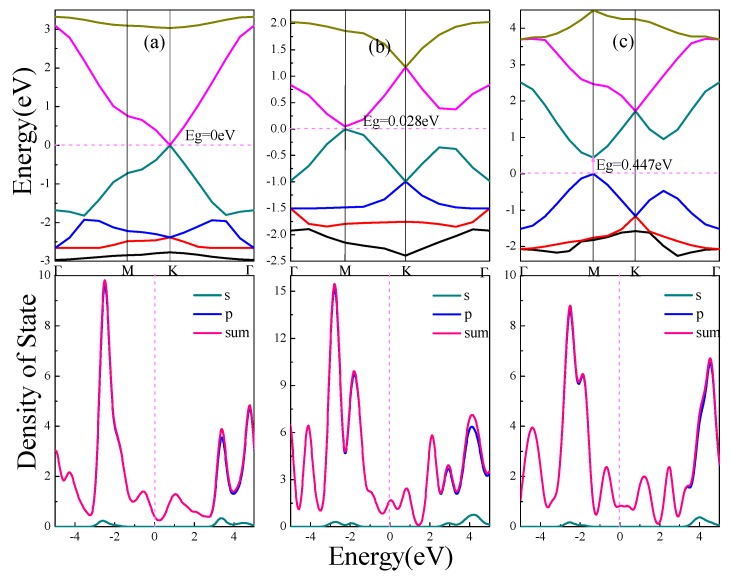
Energy band and density of states of graphynes (α, β, γ). (**a**) α-graphyne. (**b**) β-graphyne. (**c**) γ-graphyne. In this picture, Eg indicates the band gap. The letters s and p represent partial density of states of s orbital and p orbital of C atoms respectively, and sum represents total density of states.

The electronic structure of α, β, γ-graphynes are analyzed with an emphasis on the effect of strain on the band gap. Band gap represents the difference between the conduction band minimum and the valence band maximum. Figure 2 shows energy band and density of state (DOS) for the three graphyne structures.

As shown in Figure 2a for the case of α-graphyne, the conduction band minimum and valence band maximum coincide at one point on the Fermi level, which is the K point in Brillouin zone; On both sides of the Fermi level, the band gap diverges from the K point to form two symmetric Dirac cones, which has zero curvature at least in one direction. The α-graphyne has a zero band gap and DOS approaches zero at Fermi level. For β-graphyne and γ-graphyne, the energy band forms Dirac zone at point M in Brillouin zone, and direct band gaps of 0.028 eV and 0.447 eV are shown in Figure 2b,c respectively.

From the above discussion, all three graphyne structures are semiconductor materials, with no band gap for α-graphyne, and a smaller band gap for β-graphyne than γ-graphyne. In all cases the energy bands near Fermi level are wide with steep ups-and-downs. The aggregated DOS peaks at Fermi level are mild, and, as also indicated in PDOS, the energy band near Fermi level consists mostly of expansive p orbitals with significantly delocalized carriers of small effective mass. This indicates that the peculiar electronic features near Fermi level (Dirac point and Dirac cone) come from the small effective mass and large mobility of the p orbitals carriers. 

From the binding energy perspective, zero band gap α-graphyne has the smallest binding energy, and γ-graphyne with the largest binding energy has the largest band gap, whereas β-graphyne comes in between.

**Figure 3:**
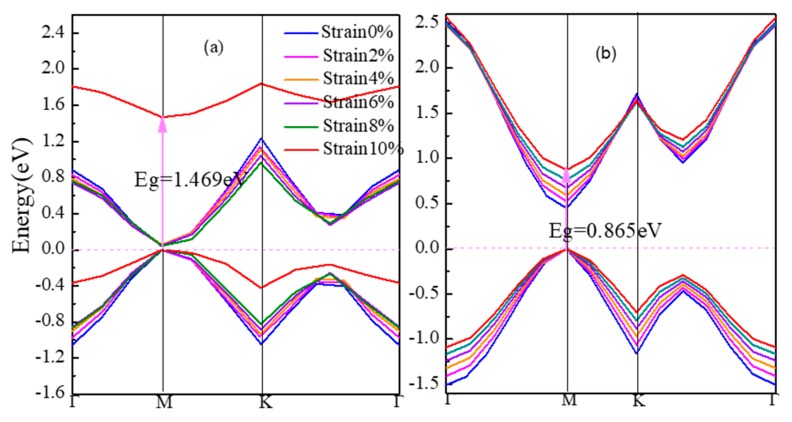
The band gap of strained graphynes (**a**) β-graphyne, (**b**) γ-graphyne.

Figure 3 shows the energy band structures of strained β-graphyne and γ-graphyne in Figure 3a, b respectively. From 0% to 10% strain, the band gap of α-graphyne remains zero. The band gap of β-graphyne does increase with strain, albeit minimally before the strain reaches 8%, where it goes up rapidly, hitting 1.469 eV at 10% strain. The band gap of γ-graphyne increases linearly with the latter from early on. For semiconductor materials, the regulation of band gap can make them to be better used in the field of optoelectronics. Opening β-graphyne’s band gap requires strong stress. However, γ-graphyne’s band gap can be regulated regularly. People can precisely tune its band gap for application as needed. Therefore, γ-graphyne can be considered as a good optoelectronic material.

Stress induces in lattice distortion and defect, which hinders the motion of carriers. This is manifested in lowered conductivity and metallicity of the material. The electronic structure of α-graphyne maintains its metallicity under stress, which again presents the small effective mass and large mobility of carriers as the cause of the Dirac point. The β-graphyne could be rather sensitive to stress when the latter reaches a significant level. The sharpened DOS peak at Fermi level at 10% strain is a sign of the localization of the p orbits and thus the weak bonds formed. The γ-graphyne has its band gap increasing linearly with strain, accompanied by nearly constant DOS, suggesting the possibility of a straightforward strain-based regulation. All these facts indicate that the defects in graphyne would make the material less of a metal and more of a semiconductor. 

From the bond nature and length listed in Table 1, there is no sp^2^-sp^2^ hybridization in the metallic, zero-band-gap α-graphyne that is insensitive to strain. In the cases of β-graphyne and γ-graphyne where the sp^2^-sp^2^ hybrid bonds do exist, the more strain-sensitive β-graphyne has longer bond length than γ-graphyne (1.456 Å vs. 1.423 Å). This shows that sp-sp hybridization serves as the stabilizer for the electronic structure in graphynes.

### 3.2. Elasticity

The material elasticity is related to crystal defects and bonds. The elastic constants of the material indicate the elastic limit of lattice under external stress [48]. In our study, ab initio calculation is used for *C_ij_*, with least squares fitting on the chosen elastic constants in the strained unite cell [49]. For the elasticity calculation of 2D material, there is only in-plane stretching or compression different from bulk materials. So, the calculated elastic constants are in [01] and [10] directions. The elastic modulus of 2D material *B*^2*D*^, Young’s modulus *E*^2*D*^ and shear modulus *G*^2*D*^ in [10] and [01] (x and y directions in the plane), and Poisson’s ratio *v* are all related to elastic constants *C*_11_, *C*_12_, *C*_22_ and *C*_66_ [50]. The various elastic parameters are shown in Table 2. Elastic modulus *B*^2*D*^ measures the film’s resistance to tensile stress. Shear modulus *G*^2*D*^ and Young’s modulus *E*^2*D*^ measure the material’s resistance to shear strain and normal stress, respectively, within the elastic limit. The Poisson ratio *v* of a 2D material represents its crystal’s stability under shear force. The smaller the ratio, the more stable it is under shear force. All these parameters represent, under some circumstance, the strength of the bond and its resistance to external forces [51]. The shear anisotropic factor A describes the elastic anisotropy by measuring the degree of anisotropy in the bonds between different atoms in the plane. When *A* is equal to 1, it means the material is elastic isotropy [52].

The elastic modulus of 2D material *B*^2*D*^ is a measure of the tensile resistance of layered material, calculated as [50]

(2) $$B^{2D}=\left(C_{11}+C_{22}+2C_{12}\right)/4$$

Young’s modulus of the 2D material *E*^2*D*^ in [10] and [01] directions (plane rigidity) are given as [50]

(3) $$E_{\left[10\right]}^{2D}=\left(C_{11}C_{22}-C_{12}^{2}\right)/C_{22},E_{\left[01\right]}^{2D}=\left(C_{11}C_{22}-C_{12}^{2}\right)/C_{11}$$

Poisson’s ratio is as following [50]

(4) $$V_{\left[10\right]}^{2D}=C_{12}/C_{22},\;V_{\left[01\right]}^{2D}=C_{12}/C_{11}$$

Shear modulus *G*^2*D*^ is as following [52]

(5) $$G_{\left[10\right]}^{2D}=\;G_{\left[01\right]}^{2D}=C_{66}$$

$$A=4C_{66}/\left(C_{11}+C_{22}-2C_{12}\right)$$

**Figure 4:**
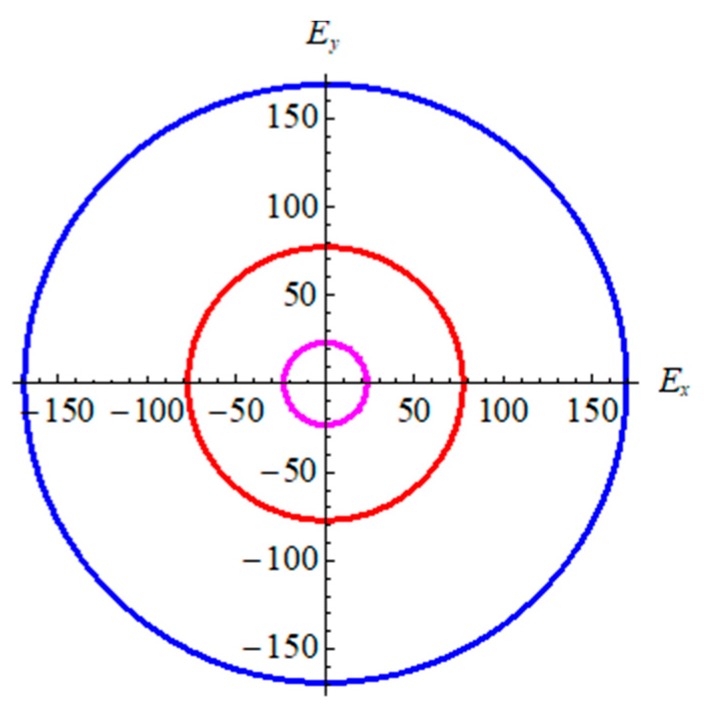
The 2D Young’s modulus for graphyne. Pink line represents α-graphyne, red line represents β-graphyne, and blue line represents γ-graphyne.

From Table 2, in terms of bond strength and binding energy, the order α-graphyne < β-graphyne < γ-graphyne stands for all mechanical parameters. This is consistent with the bond length and binding energy values in Table 1. According to the Poisson’s ratio, the value decreases gradually from γ-graphyne to α-graphyne, indicating that γ-graphyne is the most stable structure under the action of shear force, and the stability of α-graphyne is relatively weak. The above points have further demonstrated that the mechanical properties of γ-graphyne are the most excellent. In addition, all three structures are elastic isotropy, because the shear anisotropic factor A is equal to 1. In order to better reflect isotropy of three structures, two dimensional diagrams of Young’s modulus *E*^2*D*^ is exhibited in Figure 4. The formula is $E^{-1}=S_{11}cos^{4}\theta +2S_{12}cos^{2}\theta sin^{2}\theta +S_{22}sin^{4}\theta +S_{66}cos^{2}\theta sin^{2}\theta$, here *S_ij_* is the elastic compliance constants. Figure 4 shows three materials are elastic isotropy, which is consistent with the values of the shear anisotropic factor in Table 2.

### 3.3. Thermal Conductivity

The heat transfer of 2D material diverges as the distance increases [53], and the thermal conductivity decreases with increasing temperature [54]. Knowledge on the factors affecting the thermal conductivity, the minimum values under high temperature are important for their applications. Clark model is used for the calculation of the minimum thermal conductivity *k_min_* [55]. The Debye temperature *θ_D_* [56], the average acoustic wave velocity *v*_m_, average acoustic transverse and longitudinal wave velocity *v*_t_ and *v*_l_ [39] are calculated as:

(6) $$\mathrm{Clark}\;\mathrm{model:}\;k_{min}=0.87k_{B}M_{a}^{-2/3}E^{1/2}\rho^{1/6}$$

(7) $$\Theta_{D}=\frac{h}{k_{B}}{\left(\frac{3}{4\pi M}\right)}^{1/3}v_{m}$$

(8) $$v_{m}={\left(\frac{1}{3}\left(\frac{2}{{\left(v_{t}\right)}^{3}}+\frac{1}{{\left(v_{l}\right)}^{3}}\right)\right)}^{-1/3}$$

(9) $$v_{t}=\sqrt{G/\rho },v_{l}=\sqrt{\left(3B+4G\right)/3\rho }$$

where *E* is the Young’s modulus, ρ is the density, *k_B_* is the Boltzmann constant, *M_a_* = [*M*/(*n* × *N_a_*)] is the average mass of the atoms in the lattice, *M* is the molar mass, *n* is the number of atoms, and *N_a_* is the Avogadro constant. 

The minimum thermal conductivity, Debye temperature, and acoustic wave velocities of the three graphynes are shown in Table 3. That they all follow the same trend α-graphyne < β-graphyne < γ-graphyne surprises none as these quantities are all calculated from the mechanical properties with Equations (6)–(9). 

The minimum thermal conductivity *k_min_* and Debye temperature of three different graphyne structures increase in order that is consistent with their bandgap values and cell structures. Consider the number of acetylenic bonds in unit cell and bond length values in Figure 1, as well as the bandgap values in Figure 2, could see that the more acetylenic bonds in unit cell, the shorter the bond length, the larger the band gap, and the greater the thermal conductivity. In summary, the minimum thermal conductivity and Derby temperature are also related to the strength of acetylenic bonds: the stronger the bonds, the greater the thermal conductivity.

### 3.4. Optical Properties

The light absorption, which arises from the interaction between the light and the electrons and atoms of the material it passed through, reveals the nature of the material’s optical property as well as information about the electronic structure. It is usually characterized with the dielectric constant, as shown in Equation (10)

(10) $$\varepsilon \left(\omega \right)=\varepsilon_{1}\left(\omega \right)+i\varepsilon_{2}\left(\omega \right)$$

**Figure 5:**
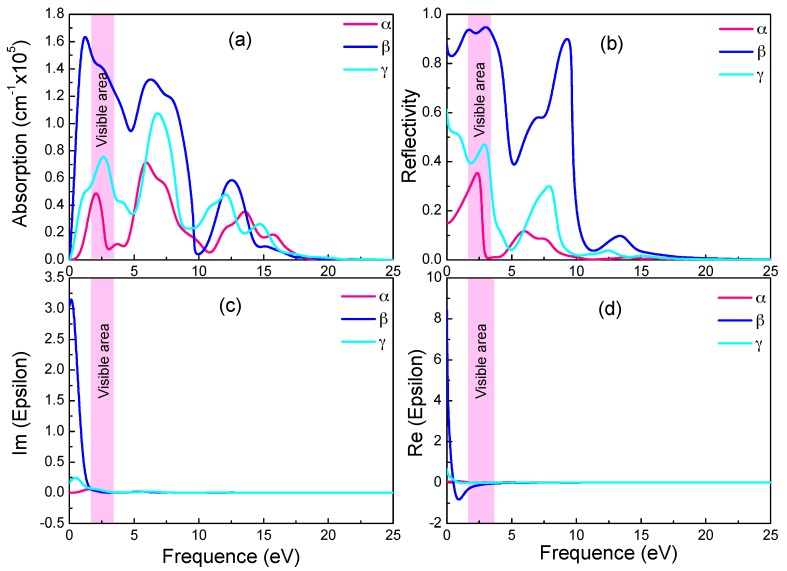
Absorption spectra (**a**), reflectivity (**b**), imaginary (**c**) and real (**d**) part of the dielectric constants of the three graphynes.

The imaginary part *ε*_2_ could be calculated directly, as it is intrinsically related to the dielectric absorption power. The real part *ε*_1_, the absorption coefficient and the reflectivity can all be deduced from *ε*_2_ with the Kramers-Kronig dispersion [57]. In Figure 5a–d give the absorption coefficient, reflectivity, imaginary and real part of the dielectric constant, respectively, as functions of photon energy for the three graphyne structures. 

In Figure 5a,c, the absorption spectra resemble the *ε*_2_ spectra in trend and peaks. Both have in near infrared and visible light ranges sharp peaks, whose intensities increase drastically with photon energy. With reference to the energy band diagram, the band shape changes abruptly in the spectrum energy range (0–3.5 eV), and indicating small electron effective mass and significant delocalization. In the absorption spectra, the absorption starts at zero photon energy, and the absorption edge energy does not correspond to relevant band gaps (0.028 eV and 0.447 eV). Since the free carrier absorption occurs at lower energy than does the intrinsic absorption, there should be significant free carrier absorption from inner-band transition in the below-the-gap energy range.

The monotonic increase of absorption coefficient in Figure 5a with energy near the visible light energy range indicates a high free carrier concentration (and small electrical resistivity). As shown in Figure 5d, the negative real part of the dielectric constant in the visible light and nearby energy range is a sign of good conductivity. In all three graphynes, the large real part of the dielectric constant (static dielectric constant) at zero photon energy is a symptom of strong polarization between electrons. The drastic drop of the β-graphyne value with the increasing energy is caused by the onset of resonance between incident photon and electron transition. There is also a minor deviation of the spectrum peak energy from the degenerated band gap, revealing the occurrence of relaxation during the transition. It also shows the electron transition energy is not a simple energy level difference.

These three graphynes possess the high reflectivity and absorption from the infrared region to visible light. The largest absorption and reflectivity in the visible light range come from the β-graphyne. 

**Figure 6:**
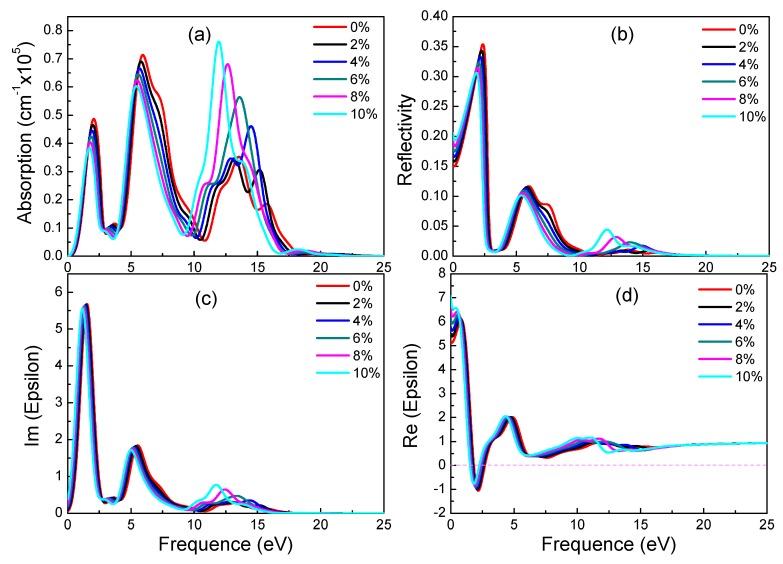
Absorption spectra (**a**), reflectivity (**b**), imaginary (**c**) and real (**d**) part of the dielectric constants of α-graphyne under different strains.

**Figure 7:**
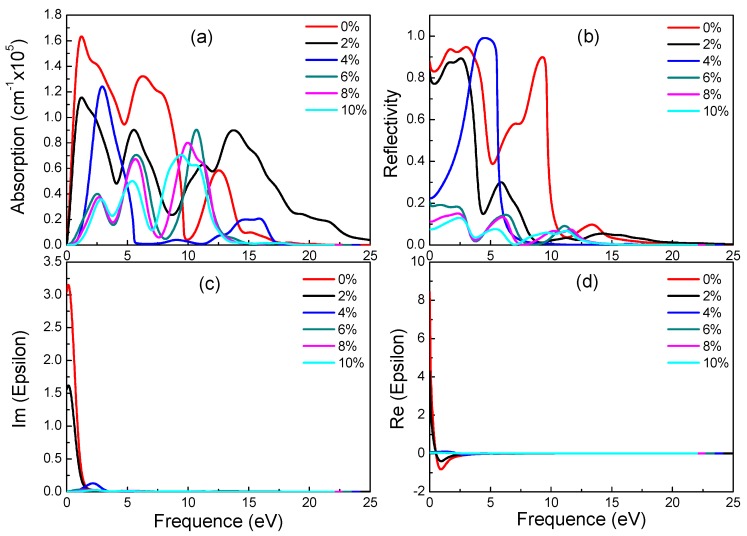
Absorption spectra (**a**), reflectivity (**b**), imaginary (**c**) and real (**d**) part of the dielectric constants of β-graphyne under different strains.

**Figure 8:**
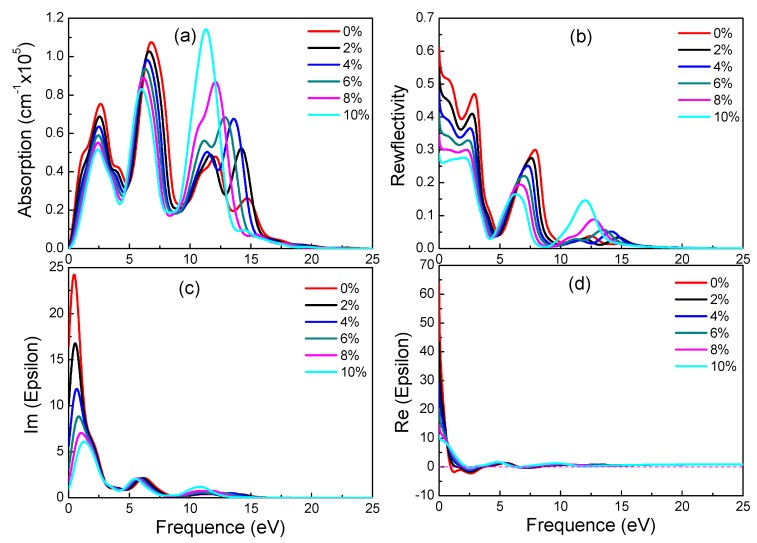
Absorption spectra (**a**), reflectivity (**b**), imaginary (**c**) and real (**d**) part of the dielectric constants of γ-graphyne under different strains.

Graphynes which have great application prospects in the field of optoelectronics are new 2D materials. However, their optical properties under strain were never studied. In the following, the influence of different strains on optical properties of three materials is discussed. Figure 6, Figure 7 and Figure 8 show absorption spectrum, reflectivity, dielectric constant’s imaginary part and real part of three graphyne under different strains respectively. 

From 0% to 10% strain, combining with electronic structures of α-graphyne, its band gaps remain zero. This phenomenon correlates well with the absorption spectrum. All absorption spectrums increase at 0 eV regardless of the applied strain. In terms of absorption, with the increase of strain, the two peaks which locate 0 eV to 10 eV decrease and the peak which locates 10 eV to 15 eV increase. In addition, the three main peaks have red-shift phenomenon. The trend of reflectivity agrees well with absorption spectrum. However, from 2% to 10% strain, the values of static reflectivity (at 0 eV) are increasing. According to Figure 6c, there is no significant change from 0 eV to 10 eV of imaginary part of dielectric constants. However, with the increase of strain, the values of peaks which locate 10 eV to 15 eV increase and there is a red-shift phenomenon. In terms of real part of dielectric constants, static dielectric constants increase gradually. These results indicate that strain can adjust optical properties of α-graphyne without band gaps.

For β-graphyne, its band gap does increase with strain, albeit minimally before the strain reaches 8%, where it goes up rapidly, hitting 1.469 eV at 10% strain. This phenomenon correlates well with the absorption spectrum. Carriers need a larger energy to transit for a larger band gap. Observing Figure 7, it is found that β-graphyne is very sensitive to strain. A small strain applied can lead to a significant change of optics. When strain reaches 2%, the peaks of absorption spectrum, static reflectivity and static dielectric constants decrease obviously. From 6% to 10%, optical properties tend to be stable. It is interesting of absorption spectrum and reflectivity to have blue-shift phenomenon. In addition, opening β-graphyne’s band gap requires strong stress. 

According to electronic structure, γ-graphyne has its band gap increasing linearly with strain. Observing Figure 8, it is found that all special values decrease linearly with increasing strain. As can be seen from Figure 8a,b, as the tensile strain increases, the peaks of reflectivity move toward a lower energy and the peak height decreases. These results suggest that optical properties of γ-graphyne can be precisely controlled by strain in engineering application.

## 4. Conclusions

Based on the first-principles, CASTEP software package is used to calculate and analyze the structural stability, electronic structure, elasticity, thermal conductivity, optical properties as well as the electronic structure and optical properties under strain of α, β, γ-graphyne. The result shows that among the three structures, γ-graphyne is the best in terms of structural stability, synthetization, elasticity, thermal conductivity, and sensitivity to strain. This is because it has in the unit cell most acetylenic bonds from sp hybridization, which has the shortest bond length, highest bond strength and binding energy. According to analysis of computation, finding the thermal conductivity is related to the band gap, and γ-graphyne with the largest band gap has the largest thermal conductivity. The band gap of γ-graphyne increases linearly with strain. Analysis on the optical properties shows that, in all three graphynes, the absorption starts at zero photon energy, and the absorption edge energy does not correspond to the relevant band gap. This is due to the free carrier absorption at energies lower than that of the intrinsic absorption, which implies that, in addition to the valence-band-to-conduction-band transition, there is also the inner-band transition near the Fermi level. There is also a minor deviation of the spectrum peak energy from the degenerated energy band, due to the relaxation on the electron transition, so that the energy absorbed is not simply equal to the band gap. In addition, strain can adjust optical properties of α-graphyne without changes of band gaps, and small strain can change optics of β-graphyne obviously, and optical properties of γ-graphyne decrease linearly with increasing strain. In this paper, strain is an effective way to modulate the electronic and optical properties of graphynes. The versatile structures of graphynes give them outstanding features for extensive application scope.

## Figures and Tables

**Table 1:** Graphyne (α, β, γ) model parameters: lattice constants *a*, *b* (Å), density *ρ*^2*D*^ (g·cm^−2^), bond type and bond length (Å), band gap (eV), total energy *E_total_* (eV), binding energy *E_coh_* (eV).

Species	Method	*a*	*ρ*^2*D*^	*sp-sp*	*sp-sp*^2^	*sp*^2^*-sp*^2^	*Band Gap*	*E_total_*	*E_coh_*
α-graphyne	GGA-PBE	6.950	2.357	1.229	1.392	-	0	−1233.384	−8.343
	GAA-PBE-D	6.948	2.359	1.228	1.391	-	0.005	−1233.57	−8.366
	Other work	6.966 ^a^, 7.01 ^b^		1.230 ^a^	1.396 ^a^	-	-	-	-
β-graphyne	GGA-PBE	9.459	2.863	1.232	1.386	1.456	0.028	−2776.578	−8.424
	GAA-PBE-D	9.454	2.867	1.231	1.385	1.455	0.04	−2777.185	−8.458
	Other work	9.47 ^c^, 9.464 ^d^		1.232 ^a^	1.389 ^a^	1.457 ^a^	-	-	-
γ-graphyne	GGA-PBE	6.875	3.614	1.222	1.403	1.422	0.447	−1853.464	−8.625
	GAA-PBE-D	6.870	3.619	1.221	1.403	1.422	0.448	−1853.99	−8.699
	Other work	6.89 ^a^,6.86 ^e^	2.357	1.223 ^a^	1.408 ^a^	1.426 ^a^	0.46 ^f^	-	−7.95 ^e^

^a^ Ref. [21]. ^b^ Ref. [44]. ^c^ Ref. [45]. ^d^ Ref. [32]. ^e^ Ref. [46]. ^f^ Ref. [47].

**Table 2:** Graphyne (α, β, γ): elastic constants *C_ij_*, shear modulus *G*^2*D*^ elastic modulus *B*^2*D*^, Young’s modulus *E*^2*D*^ (N·m^−1^) and Poisson’s ratio *v*^2*D*^.

Parameters	α-Graphyne	β-Graphyne	γ-Graphyne
*C*_11_	95	133	202
*C*_12_	82	86	82
*C*_22_	95	133	202
*C*_66_	6.5	23.5	60
*S*_11_	0.043	0.013	0.006
*S*_12_	−0.037	−0.008	−0.002
*S*_22_	0.043	0.013	0.006
*S*_66_	0.161	0.043	0.017
*G*^2*D*^	6.5	23.5	60
*B*^2*D*^	89	110	142
*E*^2*D*^_[10]_ = *E*^2*D*^_[01]_	24 (22 ^a^)	77 (73 ^a^)	169 (166 ^a^, 169 ^b^)
*v*^2*D*^_[10]_ = *v*^2*D*^_[01]_	0.863 (0.87 ^a^)	0.647 (0.67 ^a^)	0.406 (0.42 ^a^, 0.417 ^b^)
*A*	1	1	1

^a^ Ref. [21]. ^b^ Ref. [19].

**Table 3:** The minimum thermal conductivity *k_min_* [W/(m·K)], Debye temperature *θ_D_* (K), average acoustic transverse wave velocity *v_t_*, average acoustic longitudinal wave velocity *v_l_*, average acoustic wave velocity *v_m_* (km s^−1^).

Species	*k_min_*	*θ_D_*	*v_t_*	*v_l_*	*v_m_*
α-Graphyne	0.920	220	1.322	3.246	1.497
β-Graphyne	1.650	307	1.853	3.823	2.083
γ-Graphyne	2.456	410	2.274	4.082	2.532
